# The Role of Oscillatory Phase in Determining the Temporal Organization of Perception: Evidence from Sensory Entrainment

**DOI:** 10.1523/JNEUROSCI.1704-17.2017

**Published:** 2017-11-01

**Authors:** Luca Ronconi, David Melcher

**Affiliations:** Center for Mind/Brain Sciences, University of Trento, 38068 Rovereto, Italy

**Keywords:** behavioral oscillations, oscillations, temporal perception, time perception, visual perception

## Abstract

Recent behavioral, neuroimaging, and neurophysiological studies have renewed the idea that the information processing within different temporal windows is linked to the phase and/or frequency of the ongoing oscillations, predominantly in the theta/alpha band (∼4–7 and 8–12 Hz, respectively). However, being correlational in nature, this evidence might reflect a nonfunctional byproduct rather than having a causal role. A more direct link can be shown with methods that manipulate oscillatory activity. Here, we used audiovisual entrainment at different frequencies in the prestimulus period of a temporal integration/segregation task. We hypothesized that entrainment would align ongoing oscillations and drive them toward the stimulation frequency. To reveal behavioral oscillations in temporal perception after the entrainment, we sampled the segregation/integration performance densely in time. In Experiment 1, two groups of human participants (both males and females) received stimulation either at the lower or the upper boundary of the alpha band (∼8.5 vs 11.5 Hz). For both entrainment frequencies, we found a phase alignment of the perceptual oscillation across subjects, but with two different power spectra that peaked near the entrainment frequency. These results were confirmed when perceptual oscillations were characterized in the time domain with sinusoidal fittings. In Experiment 2, we replicated the findings in a within-subject design, extending the results for frequencies in the theta (∼6.5 Hz), but not in the beta (∼15 Hz), range. Overall, these findings show that temporal segregation can be modified by sensory entrainment, providing evidence for a critical role of ongoing oscillations in the temporal organization of perception.

**SIGNIFICANCE STATEMENT** The continuous flow of sensory input is not processed in an analog fashion, but rather is grouped by the perceptual system over time. Recent studies pinpointed the phase and/or frequency of the neural oscillations in the theta/alpha band (∼4–12 Hz) as possible mechanisms underlying temporal windows in perception. Here, we combined two innovative methodologies to provide more direct support for this evidence. We used sensory entrainment to align neural oscillations to different frequencies and then characterized the resultant perceptual oscillation with a temporal dense sampling of the integration/segregation performance. Our results provide the first evidence that the frequency of temporal segregation can be modified by sensory entrainment, supporting a critical role of ongoing oscillations in the integration/segregation of information over time.

## Introduction

Although sensory input is continuous, perception involves grouping information over time. In speech processing, for example, auditory signals are grouped into units ranging from phonemes (milliseconds) to words (hundreds of milliseconds) and sentences (seconds). Likewise, the visual stream is parsed into units such that, for example, two successive flashes are interpreted as simultaneous if separated by a short time interval (20–50 ms) (Hirsh and Sherrick, 1961).

Recent electrophysiological studies in humans have renewed the idea (Harter, 1967) that ongoing oscillations are linked to the intrinsic tendency of the perceptual system to process information within different temporal windows. Ongoing activity in the theta (4–7 Hz) and alpha (8–12 Hz) band EEG are major candidate mechanisms because they have been shown to correlate with various aspects of perception (VanRullen and Koch, 2003; Morillon and Schroeder, 2015; VanRullen, 2016). This relationship between ongoing oscillations and temporal windows in perception suggests two testable hypotheses. First, there should be “alignment” between behavior and the oscillation, with performance depending on the ongoing phase of the oscillation. Phase-dependent perception was demonstrated originally in EEG studies testing simple near-threshold detection or reaction times tasks (Callaway and Yeager, 1960; Busch et al., 2009; Mathewson et al., 2009; Fiebelkorn et al., 2013; Hanslmayr et al., 2013). However, more recently, a role of the ongoing oscillatory phase was also reported for tasks measuring whether two stimuli are integrated or segregated in time (Varela et al., 1981; Mathewson et al., 2012; Wutz et al., 2014, 2016; Milton and Pleydell-Pearce, 2016). Second, an oscillation with a fast/short cycle should lead to faster alterations in behavior. Recent evidence for this idea comes from a link between alpha oscillation frequency in the prestimulus interval and the two-flash fusion threshold, with individuals with faster prestimulus alpha frequency exhibiting better temporal segregation (Samaha et al., 2015).

However, more causal evidence linking perception with the phase and/or frequency of different oscillatory rhythms requires methods that manipulate neural oscillations directly. One way to test this is by exploiting the intrinsic tendency of neural oscillations to show entrainment to periodic external forces. Possible forces that can lead to entrainment include magnetic or electrical forces such as those created by transcranial magnetic stimulation/transcranial alternating current stimulation, as well as rhythmic sensory input (Thut et al., 2011). Rhythmic visual or auditory stimulation at a specific frequency can entrain neural oscillations, leading to resonance phenomena in neural and perceptual activity (Thut et al., 2011; Mathewson et al., 2012; de Graaf et al., 2013; Herrmann et al., 2016). Sensory entrainment allows a precise manipulation of ongoing oscillations immediately before the stimulus appearance, influencing neural oscillations for several oscillatory cycles afterward. Spaak and colleagues (2014), for example, provided neurophysiological and psychophysical evidence that 1.6 s of visual stimulation at 10 Hz was able to influence the detection of low-contrast stimuli for up to 3 alpha cycles after the entrainment (see also de Graaf et al., 2013).

Here, we aimed to provide more direct support linking ongoing oscillations with the temporal organization of perception and to test the flexibility of the internal rhythms of temporal organization. In Experiment 1, we used entrainment at the lower and upper boundary of the alpha band (∼8.5 vs 11.5 Hz) in two different large groups of subject (*N* = 30 each). We hypothesized that two different stimulation frequencies would both align ongoing alpha oscillations and entrain them toward either a slower or faster frequency. In Experiment 2, we followed the same logic and used sensory entrainment in a within-subject design in which we additionally tested frequencies outside the alpha band (theta: ∼6.5 Hz; beta: ∼14.5 Hz). To measure the effect of entrainment, we used a temporal “dense sampling” of the integration/segregation performance, whereby the same perceptual judgment (“one flash” vs “two flashes,” given two flashes separated by ∼40 ms) was tested at regular and densely spaced time points to measure fluctuations in perception that emerged as a consequence of the entrainment.

## Materials and Methods

### 

#### Experiment 1

##### Participants.

A total of 60 participants aged 18–30 took part in the present study as paid volunteers. They were randomly split into two groups of 30 participants each (9 males, 21 females), who received sensory entrainment at two different frequencies (8.5 and 11.5 Hz, see “Experimental design” section). They reported no history of neurological disorders or epilepsy. All of them reported normal or corrected-to-normal vision and normal hearing and gave informed written consent. The experimental protocol was approved by the University of Trento ethical committee and was conducted in accordance with the Declaration of Helsinki.

##### Apparatus and stimuli.

All visual stimuli were displayed on a 22-inch CRT screen at 1024 × 768 pixels of spatial resolution with a vertical refresh rate of 160 Hz. The auditory stimuli used for the entrainment were sinusoidal 500 Hz sounds presented through professional headphones. The visual stimuli used for the entrainment were white bars sized 17 × 2° presented at full contrast and at 10° of eccentricity from the fixation point. The target stimuli (hereafter referred to as “flashes”) were luminance-defined Gaussian blob sized at 0.5 × 0.5°. The contrast of the flashes was adjusted individually before the experiment to ensure that the stimulus was presented above threshold. We first determined with a QUEST procedure the absolute contrast threshold. The resulted contrast value was then multiplied by a factor ranging from 1.5 to 2.5 in steps of 0.5. Specifically, we chose the lowest multiplication factor (1.5, 2, or 2.5) of the absolute contrast threshold value at which participants were able to detect the stimulus (either as one flash or two flashes) in all 20 trials of the practice block in which, as in the real experiment, the entrainment stimuli were presented before the target stimuli. This procedure allowed us to ensure that, for each participant, target flashes were presented at a suprathreshold level despite the presentation of higher-contrast bars in the preceding entrainment period that in some cases could induce short-term luminance adaptation even if at a nonoverlapping spatial position. Critically, we chose to use relatively weak flashes to increase our sensitivity to the effects of ongoing oscillations. A very high-contrast stimulus might itself create a full phase reset and thus decrease the influence of prestimulus, ongoing oscillations. Conversely, the use of stimuli well above the detection threshold ensured that differences in behavior were not due to variability in detecting the stimuli themselves, but rather in the interpretation of a bi-stable stimulus as either showing one or two flashes. The experiment was programmed in MATLAB using the PsychToolbox (Brainard, 1997) and all visual stimuli were displayed on a middle gray background.

##### Experimental design.

**Figure 1.**
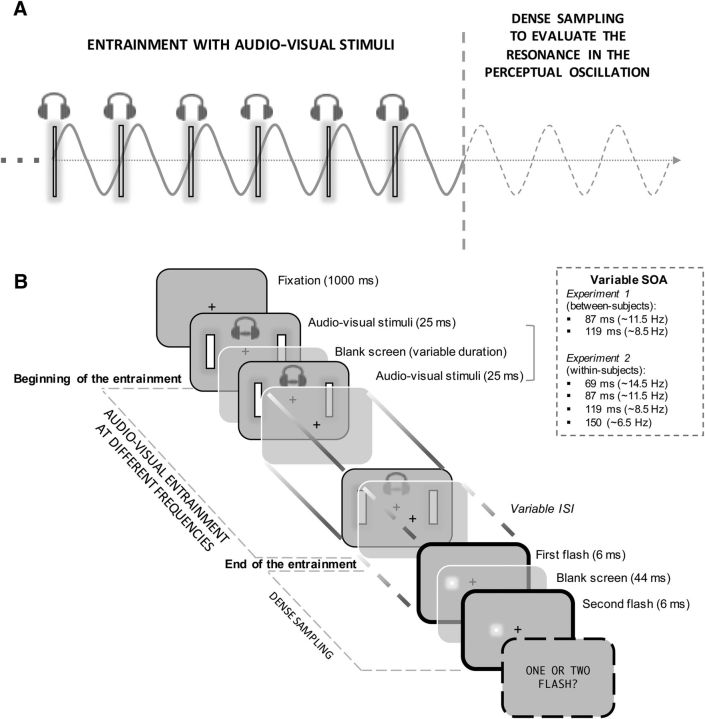
Schematic representation of the task procedure. ***A***, Main idea behind the task design. ***B***, Schematic task procedure. Participants had to report whether they perceived one or two flashes after a multisensory (audiovisual) entrainment sequence running at ∼8.5 or 11.5 Hz (Experiment 1) or at ∼6.5, 8.5, 11.5, or 14.5 Hz (Experiment 2). The ISI (interstimulus interval) between the end of the entrainment and the bi-stable stimuli presentation (i.e., two flashes that sometimes are perceived as only one) was sampled densely to reveal possible entrainment-dependent perceptual oscillations in temporal integration/segregation.

All trials started with the onset of a fixation point for 1000 ms, after which the entrainment period started (Fig. 1). The prestimulus entrainment was created with synchronized audiovisual stimuli. A combination of visual and auditory stimuli was used to maximize the effect of the entrainment. Indeed, visual and auditory events have both been shown to cause a “phase reset” of functionally relevant oscillations within (i.e., from visual stimuli to visual areas; Landau and Fries, 2012) and also across (Fiebelkorn et al., 2011; Romei et al., 2012) sensory modalities (i.e., from auditory stimuli to the visual areas). Previous evidence suggests a possible advantage of the auditory above the visual rhythmic stimulation in inducing entrainment with functional consequences in visual tasks (Ronconi et al., 2016), leading to the choice of including both visual and auditory entrainment to maximize the possibility of yielding strong temporal entrainment.

Each audiovisual stimulus combination was repeated 16 times before the onset of the target flashes. In the 11.5 Hz condition, the audiovisual entrainment stimuli were presented repeatedly for 4 refresh cycles, separated by 10 refresh cycles of blank screen. This way, the stimulus onset asynchrony (SOA) between two pairs of audiovisual stimuli in the entrainment sequence was set at 87 ms (∼11.5 Hz). In the 8.5 Hz condition, the audiovisual stimuli were presented repeatedly again for 4 refresh cycles, but separated by 15 refresh cycles of a blank screen, resulting in a SOA of 119 ms (∼8.5 Hz). The stimulus presentation methodology is illustrated in Figure 1.

After the end of the entrainment sequence, we sampled the bi-stable perception of one versus two flashes densely in time by randomly varying the interstimulus interval (ISI) between the end of the entrainment sequence (our alignment point) and the presentation of the two target flashes. This follows the logic of the dense-sampling procedure to measure the presence of behavioral oscillations by carefully measuring fluctuations in behavior over time aligned to a putative reset point (Fiebelkorn et al., 2011; Landau and Fries, 2012). To sample the integration/segregation performance densely, we used 42 regularly spaced levels of ISIs between the end of the entrainment sequence (our alignment point) and the presentation of the target flashes, with values ranging between ∼6 and 262 ms (from 1 to 42 refresh cycles). In this way, we could cover almost three alpha cycles, a range within which previous studies showed the maximal effect for sensory entrainment of similar duration (de Graaf et al., 2013; Spaak et al., 2014).

The two target flashes were displayed in the left or right hemifield at 6° of eccentricity from the fixation aligned to the horizontal axis. The side of presentation was randomized. The two flashes were always separated by seven refresh cycles (∼44 ms). This value was chosen based on pilot experiments showing that this time interval was optimal to obtain a bi-stable stimulus that, despite being physically constituted by two distinct flashes, was sometimes perceived as one flash and sometimes as two flashes.

At the end of each trial, participants were asked to report whether they perceived one or two flashes, responding at their own pace with no time constraints (Fig. 1). The total amount of trials administered for each participant was 1008, consisting of 840 bi-stable trials (20 repetitions for each ISI) and 168 “catch” trials in which only one flash was shown. The different types of trials were intermixed randomly and split into smaller blocks to prevent fatigue. The entire experimental session lasted ∼90–100 min.

#### Experiment 2

Experiment 2 was performed to confirm and extend the findings from Experiment 1 to test the frequency specificity of the effects by also including entrainment frequencies outside of the alpha band (i.e., theta and beta). Moreover, because Experiment 1 was performed following a between-subject design, we cannot completely exclude the possibility, albeit remote, that differences in the overall temporal integration/segregation performance after the entrainment were driven by an inherent difference in the endogenous alpha band activity present in the two groups. For this reason, we tested the effect of sensory entrainment at four different frequencies (theta: ∼6.5 Hz; lower alpha: ∼8.5 Hz, upper alpha: ∼11.5 Hz; beta: ∼14.5 Hz) on temporal integration/segregation performance in a within-subjects design.

##### Participants.

A total of 17 participants aged 18–30 took part in the present study as paid volunteers (7 males, 10 females). They reported no history of neurological disorders or epilepsy. All of them reported normal or corrected-to-normal vision and normal hearing and gave informed written consent. The experimental protocol was approved by the University of Trento ethical committee and was conducted in accordance with the Declaration of Helsinki.

For this group of participants (all except one), we also had the resting-state EEG data available from a previous study recorded from a 64-channel system for AC/DC recording (Brain Products). Resting data were obtained from 3 min of eyes-closed recording (online reference Cz), offline referenced to an average reference, and band-pass filtered between 0.01 and 80 Hz before extracting the FFT spectrum. From this analysis, we could confirm that the average individual alpha frequency (IAF) peak was, as expected, ∼10 Hz (mean of IAF across the channels Cz, Pz, POz, and Oz was 10.1 Hz, SD = 0.77, min = 8.9, max = 11.5). This implies that, at least for the entrainment within the alpha band, the two entrainment frequencies chosen (8.5 and 11.5 Hz) were on average at a symmetrical distance from the IAF peak.

##### Apparatus and stimuli.

All apparatus and stimuli characteristics were identical to those of the Experiment 1.

##### Procedure.

The procedure of the experiment was identical to that of the Experiment 1 except for the following changes. The entrainment sequence had four different frequencies that were obtained by changing the SOA between the audiovisual stimuli. In addition to the SOA already used in Experiment 1 (87 and 119 ms for the 11.5 and 8.5 Hz entrainment conditions, respectively), we added two other entrainment conditions. In one of the new conditions, the SOA was 150 ms (4 refresh cycles for stimuli presentation and 20 refresh cycles of blank screen), leading to entrainment frequencies of ∼6.5 Hz. In the other new condition, the SOA was 69 ms (4 refresh cycles for stimuli presentation and 7 refresh cycles of blank screen), leading to entrainment frequencies of ∼14.5 Hz.

The number of temporal dense-sampling points after the entrainment was reduced to 10, so that we sampled the one versus two flash perception at every 4 refresh cycles (from 25–250 ms in regular steps of 25 ms). The number of trials for each ISI was 22, leading to a total of 880 trials administered to each participant in the overall experiment, which lasted ∼80–90 min. Therefore, to test more entrainment frequencies within each participant, the number of samples and trials per condition were necessarily reduced, leading to some loss in the overall power of the paradigm to find any effects.

## Results

### Experiment 1

In our data analysis, we considered the two-flash rate (TFR) exhibited by participants during the presentation of the two flashes both globally and as a function of the ISI between the end of the entrainment sequence and the stimuli presentation. In the first case, we were able to test the effect of the different entrainment conditions on the general temporal integration/segregation performance, whereas in the second case, we aimed to characterize the perceptual oscillation both in terms of the main oscillatory component and phase locking across participants.

#### Overall temporal integration/segregation performance

**Figure 2.**
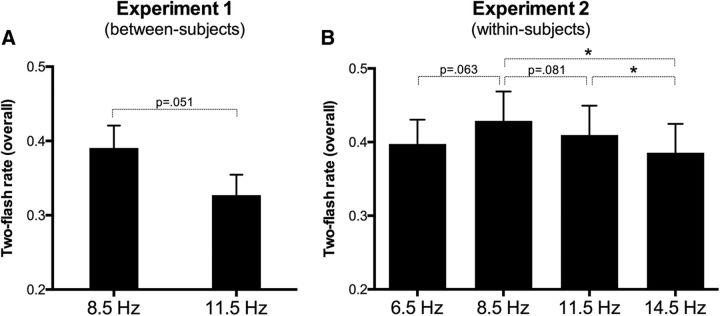
Average two-flash rate (TFR) across interstimulus intervals (ISIs) as a function of the entrainment frequency in Experiment 1 (***A***; only alpha tested, between-subjects design) and Experiment 2 (***B***; two additional frequencies outside alpha were tested, all in a within-subjects design). Error bars indicate ±1 SEM. *p < 0.05.

The raw data, expressed as the TFR averaged across participants, are depicted in Figure 2*A*. Overall, we found a tendency for a difference in the TFR exhibited across ISIs reported by two groups that, however, was in the opposite direction relative to what expected from the hypothesis of a frequency-dependent visual temporal acuity (e.g., Samaha and Postle, 2015). Indeed, the 8.5 entrainment group showed a higher, albeit only marginally significant, TFR relative to the 11.5 Hz group (11.5 Hz group: mean = 0.32, SD = 0.12; 8.5 Hz group: mean = 0.39, SD = 0.13; *t*_(58)_ = −2.00, *p* = 0.051).

#### Power spectrum of perceptual oscillations

All trials were first sorted by the ISI. For each individual, the raw data were downsampled by averaging the TFR over two subsequent ISIs (leading in total to 40 trials per temporal bin entered in the analysis for each participants). Similarly to what has been done in other previous studies measuring behavioral oscillations (Song et al., 2014), a band-pass filter (IIR order 2) between 7 and 15 Hz was applied to the raw downsampled data. After the application of zero padding to increase the frequency resolution, we calculated the individual Fourier spectrum. For each individual dataset, we calculated 1000 permutations obtained from the real data by randomizing the ISI labels. Permuted data were analyzed with the same procedure described for real data (downsampling, filtering, and zero padding) before undergoing fast Fourier transform (FFT). The amplitude values, averaged across participants, of the 1000 Fourier spectra obtained from the permutation of the real data constituted the null distribution, which was then used to test the significance of the frequency bin of interests (in the range 7–15 Hz). The final *p-*value for each frequency bin was calculated as the percentile of the observed amplitude values in the entire set of amplitude values of the permutation distribution.

**Figure 3.**
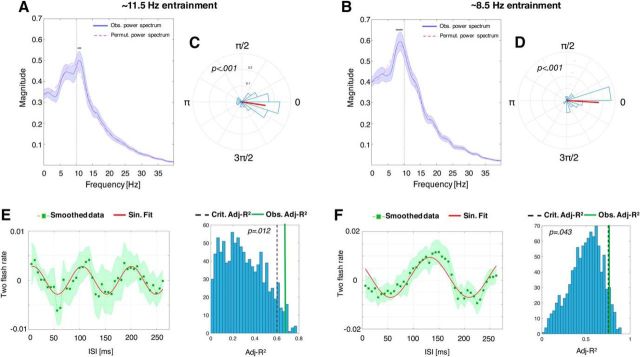
Changes in perceptual oscillations as a function of the sensory entrainment frequency within the alpha band (Experiment 1: between-subjects design). ***A***, ***B***, Power spectrum of the perceptual oscillation after the 11.5 Hz (***A***) and after the 8.5 Hz (***B***) sensory entrainment. Gray horizontal insets above the peak show the frequency bins for which the observed spectrum (average of the individual power spectrums) has been found to be significantly higher relative to the spectrum obtained from the permutation test. ***C***, ***D***, Phase angle histograms for the 11.5 and 8.5 Hz entrainment, respectively, with both perceptual oscillations that showed significant phase concentration across participants (mean phase angle vector across participants depicted in red). ***E***, ***F***, Smoothed data (i.e., moving average applied) with the shaded area representing ±1 SEM and the continuous line depicting the best sinusoidal fit in the frequency range of interest (see Materials and Methods). As evidenced from the histograms on the right, in both cases, the observed adjusted *R*^2^ (continuous vertical lines) was higher than the critical adjusted *R*^2^ value (dashed vertical line) representing the 5° percentile in the null distribution obtained from the permutation of the original data. ISI, Interstimulus interval (between the end of the entrainment and the onset of the target stimuli).

For the 11.5 Hz entrainment group, we observed an average peak in the power spectrum at 10.86 Hz, with observed values significantly higher than the permutation spectrum in the 10.5–11.6 Hz frequency range (0.012 < *p* < 0.038; Fig. 3*A*). In sharp contrast, for the 8.5 Hz entrainment group, we observed an average peak of the power spectrum at 9.05 Hz, with observed values significantly higher than the permutation spectrum in the 7.6–9.8 Hz frequency range (0.017 < *p* < 0.033; Fig. 3*B*).

An independent-samples *t* test (one-tailed) confirmed that the average frequency peaks observed in the two groups were significantly different (*t*_(58)_ = 1.69, *p* = 0.048).

Finally, we checked that filtering did not introduce unwanted distortions of the Fourier spectrum. By repeating the same preprocessing steps without filtering, we observed that the peaks of the power spectrums were equivalent to those obtained in the main analysis in which filtering was used. Specifically, the peak amplitude for the unfiltered spectrums was observed at 11.2 Hz for the faster entrainment condition and at 9.2 Hz for the slower entrainment condition.

Therefore, the main findings of the first analysis were significant behavioral oscillations and the frequency of the behavioral oscillation reflected the faster (11.5 Hz) or slower (8.5 Hz) entrainment frequency.

#### Phase concentration of perceptual oscillations induced by the entrainment

We also measured the phase concentration to provide confirmatory evidence for the presence of oscillatory fluctuations in perception. As in the power spectrum analyses, after sorting data for ISI, the raw data were downsampled and zero padded and phase was subsequently extracted from the data filtered at the frequency band of interest (7–15 Hz). Phase concentration among participants was tested separately within both entrainment groups using the Rayleigh test for nonuniformity of circular data (Fisher, 1995) as implemented in the MATLAB CircStat toolbox (Berens, 2009). Accordingly, for both entrainment groups, the phase at the peak of the power spectrum showed significant phase concentration (11.5 Hz entrainment group: *z* = 11.96, *p* < 0.001, Fig. 3*C*; 8.5 Hz entrainment group: *z* = 15.82, *p* < 0.001, Fig. 3*D*).

#### Sinusoidal fitting of the aggregate data

As a third test for the presence of behavioral oscillation at the entrainment frequencies, we measured the fit of a sinusoid to the fluctuations in behavior (Fiebelkorn et al., 2011; Spaak et al., 2014; Benedetto et al., 2016; Benedetto and Morrone, 2017). In particular, the effect of the two entrainment frequencies was assessed by fitting a sinusoidal curve to the aggregate data averaged across participants (20 trials per time bin for each participant, leading to a total of 600 trials for each temporal bin for the aggregate data) and comparing the goodness of fit obtained from the original data with a null distribution composed by the goodness of fit values obtained from 1000 permutations of the original data.

Data for each participant (both for the original and for the permuted data) were detrended and smoothed with a moving average before averaging across participants. For the permutations of the data, the ISI labels of individual data were permuted 1000 times and averaged to obtain 1000 permuted aggregate data. The 1000 measures of goodness of fit obtained from these permuted data constituted the null distribution against which we could compare the goodness of fit obtained from the original data. For both the original and the permuted data, the goodness of fit (adjusted *R*^2^, Adj-*R*^2^) for the following equation was calculated:

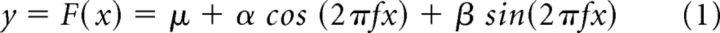
 In Equation 1, all parameters were free except for the frequency (*f*), which was constrained to the frequency band of interest (11.5 ± 2 or 8.5 ± 2 Hz). All Adj-*R*^2^ values obtained from the 1000 permuted data are plotted in the histogram in Figure 3, *E* and *F*, with the significance threshold (α = 0.05) and the Adj-*R*^2^ for the original data highlighted by the vertical lines. For both entrainment frequencies, the observed Adj-*R*^2^ was above the critical Adj-*R*^2^ (i.e., below the 5° percentile of the null distribution) obtained from the permutation procedure (11.5 Hz entrainment: Adj-*R*^2^ = .69, *p* = 0.008, best-fitting frequency = 10.6 Hz; 8.5 Hz entrainment: Adj-*R*^2^ = .75, *p* = 0.043, best-fitting frequency = 6.5 Hz). Therefore, in addition to the power and phase analyses, the sinusoid fitting analysis also provided evidence for behavioral oscillations that were significant and differed for the two different entrainment frequencies. It is worth noting that, due to the moving average used for the sinusoidal fitting of the data, which substantially acts as a low-pass filter, the best-fitting frequency is actually lower than the frequency peaks that emerged from the FFT analyses.

### Experiment 2

Using the same procedure as in Experiment 1, we analyzed the data by looking at the average TFR as a function of the four different entrainment conditions. In addition, we characterized the nature of the perceptual oscillation by testing phase locking across participants and by fitting a sinusoid to the averaged data.

#### Overall temporal integration/segregation performance

We performed a repeated-measures ANOVA having the entrainment condition as the within-subject factor (four levels: theta, lower alpha, upper alpha, and beta). The ANOVA revealed a significant effect of the entrainment condition (*F*_(3,48)_ = 2.86, *p* = 0.046; mean values were as follows: theta: mean = 0.397, SD = 0.13; lower alpha: mean = 0.429, SD = 0.16; upper alpha: mean = 0.409, SD = 0.16; beta: mean = 0.385, SD = 0.16; Fig. 2*B*). This effect was additionally explored with *post hoc t* tests (two tailed), which showed a significant difference between lower alpha and beta (*t*_(16)_ = 2.94, *p* = 0.010) and between upper alpha and beta (*t*_(16)_ = 2.46, *p* = 0.026). Nonsignificant trends were found when contrasting theta and lower alpha (*t*_(16)_ = −2.0, *p* = 0.063) and also lower alpha and upper alpha (*t*_(16)_ = 1.86, *p* = 0.081).

#### Phase concentration of perceptual oscillations induced by the entrainment

**Figure 4.**
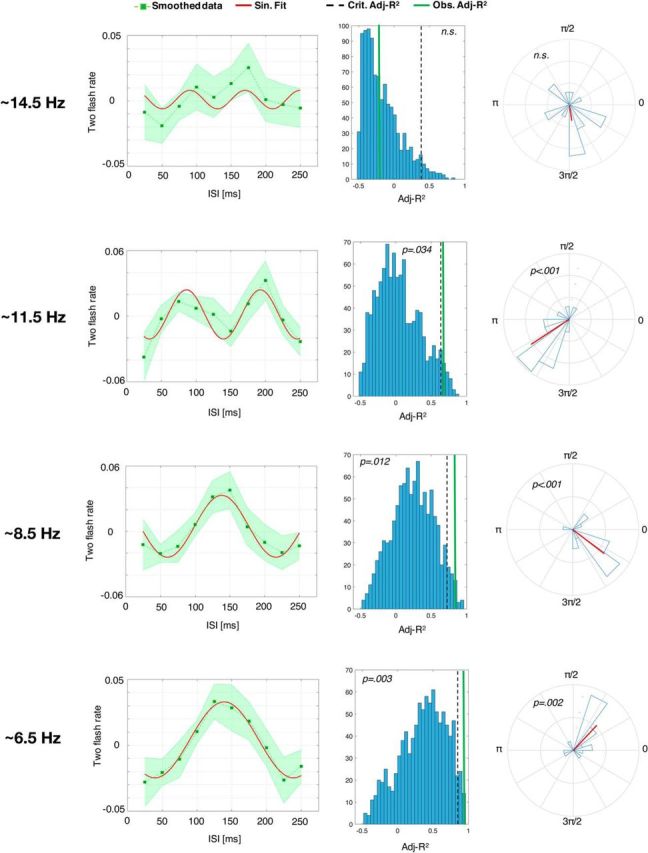
Changes in perceptual oscillations as a function of the sensory entrainment frequency within and outside of the alpha band (Experiment 2: within-subjects design). Left, Smoothed data (i.e., moving average applied) with the shaded area representing ±1 SEM and the continuous line depicting the best sinusoidal fit in the frequency range of interest (see Materials and Methods). As evidenced from the histograms on the middle column, in all cases except for the entrainment at beta, the observed adjusted *R*^2^ (continuous vertical lines) was higher than the critical adjusted *R*^2^ value (dashed vertical line) representing the 5° percentile in the null distribution obtained from the permutation of the original data. Right, Phase angle histograms for all the entrainment frequencies with all perceptual oscillations except the one after entrainment at beta, showing significant phase concentration across participants (mean phase angle vector across participants is depicted in red).

The Rayleigh test for nonuniformity of circular data revealed that there was a significant phase concentration across participants after the entrainment at lower alpha (*z* = 7.94, *p* < 0.001), upper alpha (*z* = 8.51, *p* < 0.001), and high theta (*z* = 6.01, *p* = 0.002) (Fig. 4). Conversely, the entrainment at beta did not result in a significant phase alignment of perceptual oscillations across subjects (*z* = 1.67, *p* = 0.18).

#### Sinusoidal fitting of the aggregate data

As in Experiment 1, we fit a sinusoid to the aggregate data (after detrending and smoothing the individual data with a moving average), which were obtained in this case with 22 trials per time bin for each participant, leading to a total of 374 trials for each time bin for the aggregate data. This analysis revealed that, for theta, lower alpha, and upper alpha, the observed Adj-*R*^2^ obtained from the best-fitting procedure was significant (i.e., below the 5° percentile of the null distribution derived from the permutation tests) for the entrainment at theta (Adj-*R*^2^ = .95, *p* = 0.003, best-fitting frequency = 5.02 Hz), lower alpha (Adj-*R*^2^ = .85, *p* = 0.012, best-fitting frequency = 6.5 Hz) and upper alpha (Adj-*R*^2^ = .67, *p* = 0.034, best-fitting frequency = 9.5 Hz) (Fig. 4). These results replicate the findings from Experiment 1 in a within-subject design and extend them to lower frequencies outside of the alpha band.

Conversely, the observed Adj-*R*^2^ for the beta entrainment was not significant (Adj-*R*^2^ = −0.22, *p* = 0.49; best-fitting frequency = 12.6 Hz), suggesting the absence of a perceptual oscillation at this faster entrainment frequency.

## Discussion

There is an increasing body of evidence that links the phase and/or frequency of the ongoing (prestimulus) neural oscillations to the subsequent integration or segregation of upcoming sensory information over time (for recent reviews, see Morillon and Schroeder, 2015; VanRullen, 2016). Here, we investigated directly the relationship between specific oscillations and the temporal integration/segregation of sensory events over time by performing two experiments using sensory entrainment at different frequencies within and outside of the alpha band.

Experiment 1 demonstrated that entrainment in the prestimulus period resulted in oscillations in perceptual performance for a temporal integration/segregation task. By testing two distinct frequencies at the opposite edges within the alpha band (8.5 vs 11.5 Hz), we were able to show that the frequency of the perceptual oscillation changed as a function of the entrainment frequency. By aligning the phase of neural oscillations to an external sensory rhythmic flow lasting for only ∼1 s, we were able to influence the integration/segregation of events over time. First, we found that both entrainment frequencies led to a significant phase alignment of the perceptual cycles across subjects. In addition, the two spectral profiles were shifted toward the entrainment frequency, namely ∼9 Hz for the 8.5 Hz entrainment and ∼11 Hz for the 11.5 entrainment, with the average peak of the power spectrum that was significantly different in the two groups. Therefore, our results provide strong evidence that sensory entrainment can both align and modify fluctuations in behavior that are oscillatory in nature. The different oscillatory pattern between the two entrainment groups was evident, not only in the analysis of the frequency spectrums, but also when fitting a sinusoid to the aggregate data. This pattern of results, confirmed using several converging measures, confirms that measuring behavioral oscillations is a reliable method to study oscillatory brain activity (Fiebelkorn et al., 2011; Landau and Fries, 2012; Song et al., 2014; Spaak et al., 2014; Drewes et al., 2015; Dugué et al., 2015; Benedetto et al., 2016; Benedetto and Morrone, 2017) complementary to M/EEG.

It is interesting that, for both entrainment frequencies, we observed a peak of the frequency spectrum that was shifted toward the central frequency of the alpha band (i.e., 10 Hz) relative to the stimulation frequency used. This observation implies a role for the preexisting alpha frequency, which is likely to be ∼10 Hz on average for healthy adult participants (Klimesch, 1999). One possibility is that the manipulation of oscillatory activity by means of a rhythmic exogenous force is constrained to the main endogenous frequency. This would have two potential implications. First, it suggests that the endogenous alpha frequency is involved in the present task, consistent with the long-standing claim that alpha phase is involved in organizing (integrating or segregating) stimuli that are presented sequentially with a short delay (Bishop, 1932; Callaway and Layne, 1964; Varela et al., 1981; Mathewson et al., 2012; Wutz et al., 2014, 2016; Milton and Pleydell-Pearce, 2016). One possible neural substrate would be the thalamocortical alpha because thalamic nuclei (particularly the lateral geniculate) are known to play an important role in driving cortical alpha activity (Hughes and Crunelli, 2005; Klimesch, 2012). Second, it argues for some ability to modify, via sensory stimulation but perhaps also via top-down modulation (Arnal and Giraud, 2012; Samaha et al., 2015), this oscillatory activity with consequences for perceptual processing.

In Experiment 2, we replicated the findings of Experiment 1 in a within-subject design and extended them to lower frequencies outside of the canonical alpha range (i.e., 8–12). Indeed, we found that also sensory stimulation within the high theta rhythm (∼6.5 Hz) was able to resonate in the subsequent perceptual oscillation and showed phase alignment across subjects, indicating an effective entrainment with this rhythm. Conversely, the sensory stimulation within the beta rhythm (∼14.5 Hz) did not show evidence of an effective entrainment of perceptual oscillations.

Collectively, these findings provide support for a direct role of theta/alpha oscillations and are congruent with EEG evidence showing that two stimuli always separated by the same temporal interval are more likely to be integrated together when they are presented at one phase of the theta/alpha oscillation, whereas they are more likely to be segregated when they are presented at the opposite phase (Callaway and Layne, 1964; Varela et al., 1981; Wutz et al., 2014; Milton and Pleydell-Pearce, 2016). They are also in agreement with the idea that theta/alpha phase reflects fluctuations in excitability that can periodically modulate the processing of information in the visual system (Jensen et al., 2012; VanRullen, 2016), possibly through coupling with the activity in the higher frequency gamma band (Bonnefond and Jensen, 2015). In the specific case of visual perception, mounting evidence shows that the neural sources underlying the modulation of the ongoing theta/alpha phase are linked to the activity of the posterior parietal cortex (PPC), predominantly of the right hemisphere (van Dijk et al., 2008; Thut et al., 2011; Hanslmayr et al., 2013; Jaegle and Ro, 2014). In particular, Hanslmayr et al. (2013), using simultaneous EEG-fMRI, showed that the phase of prestimulus oscillation predicts, not only the perceptual performance, but also the bidirectional information flow between the occipital cortex and right PPC (intraparietal sulcus), suggesting that the phase of brain oscillations reflects the periodic gate of visual perception by opening transient time periods in which long-distance cortical information transfer takes place (Hanslmayr et al., 2013).

Although our current findings provide clear support for a role of theta/alpha phase in segregating/integrating events over time, we found mixed evidence concerning the intriguing question of whether manipulation of the frequency of the ongoing oscillations can affect the temporal acuity of visual perception consistently impact, making people effectively “faster” or “slower.” Experiment 1 showed a marginally significant difference in the average TFR between the two groups, but this difference was in the opposite direction relative to what expected from previous studies (Samaha and Postle, 2015; see also Cecere et al., 2015). Samaha and Postle (2015) reported EEG evidence that higher prestimulus instantaneous alpha frequency was predictive of correct two-flash segregation. According to their results, we should have found a higher TFR (across ISIs) in the 11.5 Hz entrainment compared with the 8.5 Hz entrainment. Conversely, data from Experiment 2 provided evidence that entrainment at both lower and upper alpha led to an increase (albeit only a tendency for significance) in the overall TFR relative to the entrainment at theta. This result seems to be effectively in the direction hypothesized from previous study (Samaha and Postle, 2015; see also Cecere et al., 2015). One possibility is that variability in the alpha frequency during the task may have masked any effects. Another possibility is that the use of the same, specific probe for testing temporal integration/segregation might have influenced the results. The total duration of the two flashes and the interstimulus interval for the bi-stable stimulus was 56 ms, which corresponds to half of an alpha cycle at 8.9 Hz. This would make ideal for segregation an ongoing rhythm in the lower alpha band (e.g., the 8.5 Hz entrainment condition) and less ideal for segregation an ongoing rhythm at slower (e.g., theta) or higher frequencies (e.g., upper alpha and beta). The finding that the stimulus duration that leads to bi-stable perception happens to be ∼1/2 of an alpha cycle has been noted in the literature for some time (Bishop, 1932) and led to the suggestion that alpha determines, at least in part, the temporal resolution of perception.

In sum, by using rhythmic sensory entrainment, we demonstrated a link between the phase of the ongoing oscillations and the temporal organization of visual perception, specifically to the integration versus segregation of stimuli over time into coherent percepts. It remains to be clarified whether temporal perception is effectively structured into discrete windows, an idea first proposed by Stroud (1956) and later renewed on the basis of the EEG findings by Varela et al. (1981). According to this theory, the alpha cycle might relate to a discrete temporal window, which determines whether two events are bound (if they fall within the same window) or parsed (if they arrive across two adjacent windows). Alternatively, variations of neural excitability reflected in the theta/alpha oscillation phase might be sufficient to account for these results without requiring the hypothesis of a temporal window (for discussion, see Milton and Pleydell-Pearce, 2016). Specific phases associated with increased excitability at onset of the first target would promote improved processing regarding both targets (and their temporal interval). Instead of assuming that all stimuli that occur within an excitable phase period are temporally bound, this account suggests that they would be more likely to be perceived in their “real” nature as two distinct events. In contrast, phases associate with less neural excitability could be related to a relatively general “poor” quality of processing, which ultimately leads to a less clear perceptual experience. Future studies involving an active manipulation of the oscillatory activity and a precise characterization of the induced the perceptual oscillation can further clarify this question.
